# Forensic Dating of Venous Thromboembolism: Advances in Histological, Immunohistochemical and Molecular Markers

**DOI:** 10.3390/diagnostics15243211

**Published:** 2025-12-16

**Authors:** Elena Forzese, Vincenzo Cianci, Daniela Sapienza, Ludovica Pepe, Vincenzo Fiorentino, Antonio Ieni, Patrizia Gualniera, Alessio Asmundo, Cristina Mondello

**Affiliations:** 1 Department of Biomedical and Dental Sciences and Morphofunctional Imaging, Section of Legal Medicine, University of Messina, Via Consolare Valeria, 1, 98125 Messina, Italy; elena.forzese.94@gmail.com (E.F.); daniela.sapienza@unime.it (D.S.); patrizia.gualniera@unime.it (P.G.); alessio.asmundo@unime.it (A.A.); 2 Department of Human Pathology in Adult and Developmental Age “Gaetano Barresi”, Section of Pathology, University of Messina, 98125 Messina, Italy; ludopepe97@gmail.com (L.P.); vincenzo.fiorentino@unime.it (V.F.); antonio.ieni@unime.it (A.I.)

**Keywords:** thrombus age estimation, venous thromboembolism, VTE, histopathological staging of thrombosis, forensic pathology

## Abstract

Venous thromboembolism (VTE), comprising deep vein thrombosis (DVT) and pulmonary embolism (PE), is a major cause of sudden death and a frequent finding in forensic practice. Correctly estimating thrombus age is crucial to reconstruct the temporal relationship among clinical events, therapeutic decisions, and death, and also to distinguish unavoidable complications from possible diagnostic or management errors. This narrative review summarizes current macroscopic, histological, immunohistochemical, and molecular criteria for thrombus dating, integrating evidence from autopsy studies, experimental models, and clinical research. Because of the inherent heterogeneity of thrombi and postmortem changes, classical morphology frequently does not provide sufficiently precise timing, although it does allow a broad distinction between acute, subacute, and chronic thrombi. A more precise temporal characterization has recently been made possible by the introduction of immunohistochemical and molecular markers, such as neutrophils and NETs, macrophage markers, fibrinolytic system components, metalloproteinases, inflammatory cytokines, autophagy markers, aquaporins, and pro-resolving pathways. In order to improve diagnostic accuracy, a combined evaluation of these characteristics seems promising in differentiating clinically relevant time windows (within 3 days, 3–10 days, 10–21 days, and >21 days). Molecular profiling combined with advanced histopathology may eventually enable more consistent and repeatable thrombus dating standards and enhance the forensic assessment of VTE-related fatalities.

## 1. Introduction

Venous thromboembolism (VTE) represents a vascular disease that includes two clinical conditions, known as deep vein thrombosis (DVT) and pulmonary embolism (PE). Although they share the same pathogenesis, they may range from clinically silent emboli to sudden, massive, fatal embolism [1,2]. Thus, VTE can be included among the most common cardiovascular diseases, being associated with an increased global mortality, representing one of the leading causes of cardiovascular death worldwide [3,4,5].

From a pathophysiological point of view, three main factors, commonly identified as “Virchow’s triad”, are predisposed to thrombus formation: (i) endothelial damage; (ii) stasis or turbulence of blood flow; and (iii) blood hypercoagulability [6]. The first etiopathogenetic step of PE is usually represented by proximal DVT, which can cause the detachment of thrombotic fragments with subsequent embolic migration into the pulmonary circulation and partial or complete interruption of blood flow. Single or combined risk factors contribute to thromboembolism; those related to the patient (advanced age, obesity, pregnancy, infectious diseases, decompensated heart disease, varicose veins, malignancy, thrombophilia), to specific traumatic events (fractures of the pelvis and lower limbs), and to particular clinical “circumstances” at risk (prolonged bed rest, surgical and orthopedic procedures) [5,7].

Thrombi are traditionally divided into two types: white and red. White clots, or mural thrombi, are composed primarily of platelets and fibrin and often overlay damaged areas of arterial walls or cardiac chambers; in contrast, red clots, composed mainly of erythrocytes and fibrin, are more often found in the venous circulation, where blood flow is slower [8]. The estimated annual incidence of VTE varies between countries and in Italy is around 1–2 cases per 1000 inhabitants [9]. The annual incidence of pulmonary embolism is reported as 0.41–0.55 cases per 1000 persons/year [10]. The EPI-GETBP study, conducted in a French population of 342,000 inhabitants, reported DVT rates of 1.24 per 1000 persons/year and PE rates of 0.60 per 1000 persons/year, with an overall VTE rate of 1.83 per 1000 persons/year [11].

Since 2000, age-standardized mortality related to PE has steadily decreased in both sexes and in all European subregions, though not in Central Asia. Possible explanations include improved prevention strategies for VTE in hospitalized patients, more accurate thromboembolic risk stratification based on clinical history and risk factors, greater sensitivity and specificity of diagnostic tools, and advances in the acute management of PE, including more effective interventional procedures, the introduction of low-molecular-weight heparins, careful monitoring of vitamin K antagonists, progressive refinement of risk-stratified treatment strategies, and more efficient referral of patients to specialized centers [12].

Despite this overall downward trend, underestimation of mortality related to PE and thromboembolic phenomena in general cannot be ruled out. Diagnosis may be overlooked, particularly in sudden and/or out-of-hospital deaths and in individuals over 60 years of age, in whom autopsies are rarely performed [12,13]. Early diagnosis of PE may be hindered by the frequent non-specificity of symptoms and signs and by the possible asymptomatic nature of thromboembolism, which may be discovered incidentally or at a late stage. In some cases, the first clinical manifestation of PE may be sudden and unexpected death [2,14,15]. It is therefore important to emphasize that acute PE may itself represent a plausible underlying cause of death even in patients in whom no comorbidities or risk factors are identified, and it may occur under physiological conditions (e.g., pregnancy) or complicate elective surgical procedures characterized by low baseline mortality [12].

Attributing death to thromboembolic phenomena is thus often problematic and may become a real diagnostic and therapeutic challenge. Even today, the lack of symptom specificity and the fact that PE can occur in both otherwise healthy subjects and patients with pre-existing disease explain the difficulty in formulating a correct diagnosis, with numerous cases of overdiagnosis and underdiagnosis [4,16,17]. In autopsy series of sudden and unexpected deaths, the accuracy of antemortem diagnosis of DVT and PE has been reported to range between 10% and 30%, making thromboembolism one of the most frequently missed diagnoses in this setting [2,14,15,18].

In this context, given that clinical–pathological discrepancies remain common (46.8–75%) and technological advances have not necessarily been accompanied by proportional improvements in diagnostic accuracy, autopsy is still considered the diagnostic gold standard [2,13,14,15,18]. Several studies have evaluated discrepancies between clinical and autopsy diagnoses [4], and in this framework the possibility of dating the thrombus becomes particularly relevant for the assessment of potential professional errors. Indeed, the aforementioned in vivo diagnostic difficulties have led to a gradual increase in complaints and litigation for alleged medical malpractice [1,2,13,14,15,18].

In forensic settings, it is therefore of great importance to establish the causal relationship between DVT and recent events, such as trauma or medical treatments. Regarding the assessment of alleged professional liability, it is then necessary to understand whether fatal DVT/PE represented an unavoidable complication or the consequence of diagnostic or therapeutic errors. For example, post-surgical DVT cannot be automatically labeled as the result of medical error [2,4,19]. It may be related to malpractice in the event of misdiagnosis or failure to implement prophylaxis in selected patients; however, even when prophylaxis, careful diagnostic monitoring and adequate therapy are correctly implemented, they may reduce but not completely eliminate the risk of this insidious condition.

Focusing on DVT–PE misdiagnosis, the literature shows that the line between simple complication and malpractice is often blurred. Fewer than 25% of patients with acute DVT–PE present with the classic triad of dyspnea, chest pain, and hemoptysis; the early stages can be entirely asymptomatic, and up to 25% of patients with acute DVT–PE die suddenly without any appreciable clinical syndrome. In the clinical setting, only about one-third of DVT cases are successfully diagnosed, and it is estimated that DVT is the terminal cause of death in 5–10% of all hospitalized patients [2,4,19].

The rationale behind estimating the time of onset of DVT and PE is therefore to establish the causal relationship between patients’ predisposing factors, medical treatment, the thromboembolic event, and death [2,18], placing forensic science at the service of best clinical–diagnostic–therapeutic practice in the living subject. In the event of suspected medical error, only careful analysis by an expert forensic pathologist can reliably distinguish between an unavoidable complication and a true medical error [19]. To this end, many authors agree on the need for close integration of histological, histochemical, immunohistochemical, and immunofluorescent techniques [2,15,20,21,22,23,24].

The aim of this narrative review is therefore to provide a comprehensive and up-to-date synthesis of blood clot dating in forensic medicine, analyzing the following: (i) macro- and microscopic criteria and autopsy protocols; (ii) histopathological criteria for thrombus “phases”; and (iii) immunohistochemical panels with their markers, temporal patterns, and interpretative cut-offs.

## 2. Materials and Methods

### 2.1. Sources and Search Approach

The search was conducted by making use of the PubMed and Scopus database, up to 7 September 2025. We used combinations of keywords related to venous thromboembolism and thrombus dating, including (“venous thrombosis” OR “pulmonary embolism” OR “thrombus”) AND (“age” OR “dating” OR “organization”) AND (histology OR immunohistochemistry) AND (“forensic” OR “postmortem” OR “autopsy” OR “autopsy findings” OR “cadaver clots” OR “postmortem clots”).

In addition, the authors manually screened the reference lists of key forensic histopathology textbooks and of highly cited articles on thrombus age estimation, in order to identify further relevant contributions.

### 2.2. Study Selection and Data Synthesis

Because of the narrative nature of this work, a judgment-based study selection was conducted. The authors identified articles analyzing (i) autopsy series and forensic studies addressing the histological or immunohistochemical evolution of thrombi, (ii) experimental animal models (e.g., stasis-induced deep vein thrombosis) with clearly defined time points from thrombus induction, (iii) reports proposing specific temporal windows, cut-offs, or interpretative schemes for thrombus age estimation, and (iv) systematic reviews and reference texts in forensic histopathology that synthesize or critically appraise the above evidence.

Within these broad criteria, studies were included when they provided at least a description of macroscopic, histological, and/or immunohistochemical features of thrombi in relation to time, and/or explicit discussion of the forensic applicability of the proposed markers or staging systems.

Study selection and data extraction were performed in duplicate, but the authors did not systematically record the total number of records retrieved, screened, excluded, or included, and therefore a PRISMA flow diagram is not provided. Likewise, the authors did not conduct a formal quantitative assessment of study quality or risk of bias. Despite that, methodological robustness and clinical–forensic relevance were considered when weighing and discussing individual contributions.

For the purposes of this narrative review, from each included article the authors evaluated if the studies were performed on human or animal models, the main histological, immunohistochemical, or molecular markers examined, the proposed temporal pattern of expression, any suggested cut-offs or time windows, and the main strengths and limitations highlighted by the authors. These elements were then organized into three main domains: (i) autopsy and macroscopic findings, (ii) conventional histology and morphologic staging, and (iii) immunohistochemical and molecular markers.

## 3. Autopsy, Macroscopic Findings, and Histological Evidence of Thrombi

The analysis of the selected literature allowed us to outline a detailed and layered picture of current knowledge on blood clot dating.

### 3.1. Autopsy and Macroscopic Findings

In autopsy practice, as already widely documented, the diagnosis of thrombosis or embolism is both frequent and often underestimated during life; defining the timing of the thrombus—acute, subacute, or chronic—is the element that transforms a morphological description into a probative assessment. For this reason, autopsy studies insist on the need for a systematic and rigorous approach to the search for and documentation of any thromboembolic findings. The sample must include the entire thrombus, with multiple sections, since its structure is intrinsically heterogeneous, and the degree of organization can vary significantly between different regions of the same clot [25,26,27,28].

From a macroscopic point of view, it is crucial to correctly distinguish a postmortem clot from a viable thrombus. Postmortem clots appear shiny, soft, not adhered to the vascular wall, and easily removable; conversely, viable thrombi adhere to the vessel, and have an opaque surface [28].

Authors such as Micallef et al. [18] and Ro et al. [15] have emphasized that the diagnosis of pulmonary embolism at autopsy cannot ignore a thorough dissection of the main and segmental branches of the pulmonary arteries, with longitudinal opening and direct inspection of the vascular lumens. The investigation must also systematically extend to the deep veins of the lower limbs, the pelvis, and the inferior vena cava, using book sections and multiple sampling, to avoid overlooking mural or partially organized thrombi.

Many authors have documented a strong correlation, found at autopsy, between PE-related mortality and right ventricular dysfunction [6,29]. In the work of Anteby et al. [6], confirmed by Margiotta et al. [19], it was observed that patients with normal blood pressure but right ventricular dysfunction had a PE-related mortality of 9.3%, while in subjects with normal right ventricular function the mortality was 0.4%. In cases of acute right ventricular failure due to volume overload, the most frequently observed morphological alterations were right ventricular dilatation (92% of cases) and interventricular septum displacement (98% of cases), in a series of 60 deaths due to fatal pulmonary embolism. A total of 70% of the subjects also had right ventricular hypertrophy, which was correlated with a history of chronic pulmonary hypertension. The degree of hypertrophy also correlated with organized thrombi in the pulmonary artery, with a trend of overlapping with the diameter of right ventricular cardiomyocytes.

**Figure 1:**
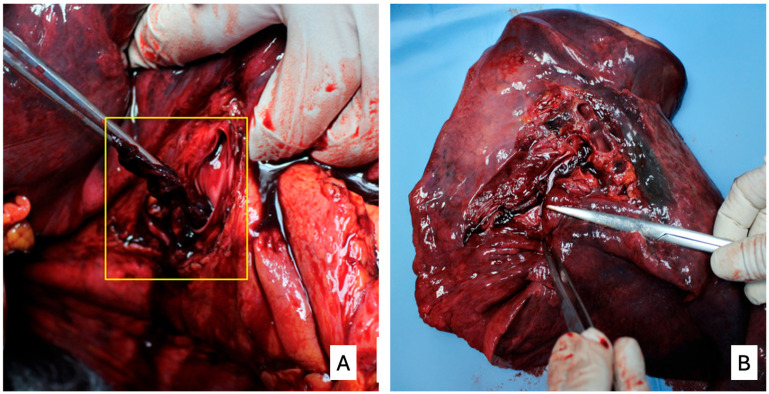
Pulmonary thromboemboli. (**A**) large spiral thromboembolus observed at the left hilum dissection (in yellow square); (**B**) thromboembolus observed in an arterial main branch after longitudinal opening of the vascular lumen.

The most macroscopically specific finding in cases of death from acute pulmonary embolism is a saddle embolus in the bifurcation of the pulmonary artery or large spiral thromboemboli (Figure 1A), often characterized by venous valve impressions confirming their embolic origin. Reddish-black emboli are easily identifiable in the main and segmental pulmonary arteries (Figure 1B), while white, organized thrombi—remnants of more recent thrombi in the process of organizing—are observed in the subsegmental arteries and small elastic vessels, taking on a “banded” or “spiderweb” appearance. Similar findings can also be found in deep veins, depending on the organization of the thrombus. The identification of these organized thrombi often allows the detection of previous and latent thromboembolisms in deceased subjects subsequently subjected to autopsy.

Following vascular occlusion caused by the thrombus, the lung parenchyma appears pale due to ischemia. This finding has also been described by Cecchi, Fineschi, and Maffeis [13,14,29]. Pulmonary infarction, however, is a rare finding, present in only 21% of cases of massive PE, likely due to double pulmonary perfusion and the short interval between onset and death [6]. Although potentially any systemic vein can originate pulmonary emboli, over 90% of lethal emboli originate from the deep veins of the lower limbs [6].

The systematic recognition of thromboemboli therefore requires the adoption of stringent autopsy protocols, especially in cases of suspected death from PE. These protocols include a detailed examination of the deep veins of the lower limbs, including not only the vessels of the thigh but also the veins of the leg, with an increase in the detection rate of residual emboli from 51% to over 90% [4,6]. However, several authors point out that the absence of thrombi in these sites does not exclude their local origin, since detachment and embolization may not leave residue [14].

In the past, acute pulmonary embolism and the chronic form—known as chronic thromboembolic pulmonary hypertension (CTEPH) [30,31]—were considered distinct pathologies. Most cases of acute PE are characterized by the presence of a large, potentially fatal thromboembolus. However, as already observed by Luijten [32], a careful examination of fixed lungs after autopsy and, above all, a detailed histopathological analysis allows for the identification of small emboli in the distal pulmonary vessels, often in the process of organizing. Dunnill defined them as “heraldic emboli,” interpreting them as residues of previous, latent thromboembolic episodes.

Subsequently, Anteby et al. [6] introduced the concept of acute-on-chronic pulmonary thromboembolism to describe the recurrence of latent thrombotic events, responsible for a progressive thrombotic occlusion in both the deep veins and the pulmonary arteries. The same authors also observed that the severity of PE, measured on the basis of the distribution of organized thrombi, correlates with the severity of deep vein thrombosis and with right ventricular dysfunction [6].

### 3.2. Histological Findings: Timeline of Thrombus “Evolution”

**Figure 2:**
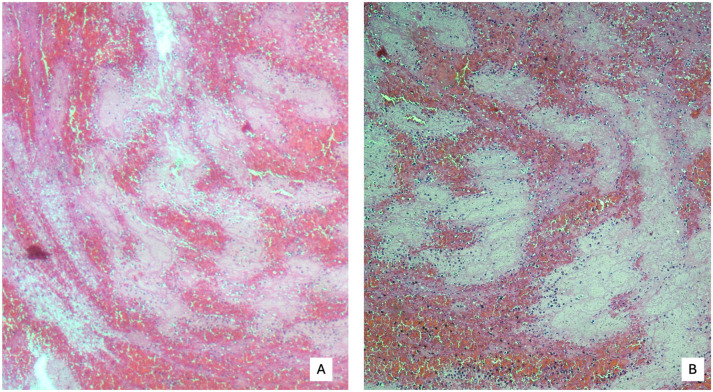
Zahn’s lines. The red bands consist of clusters of red blood cells with a few white blood cells (4× magnification (**A**)), while the pink bands represent the lines of Zahn, made up of fibrin and platelet aggregates (10× magnification (**B**)).

From a histological point of view, firstly, it is of critical importance to distinguish antemortem thrombus (AMT) from postmortem clot (PMC). To this aim, the so-called lines of Zahn, serpiginous bands of nested platelets wrapped with fibrin, are considered highly sensitive and specific microscopic features of AMT (Figure 2) [28].

**Figure 3:**
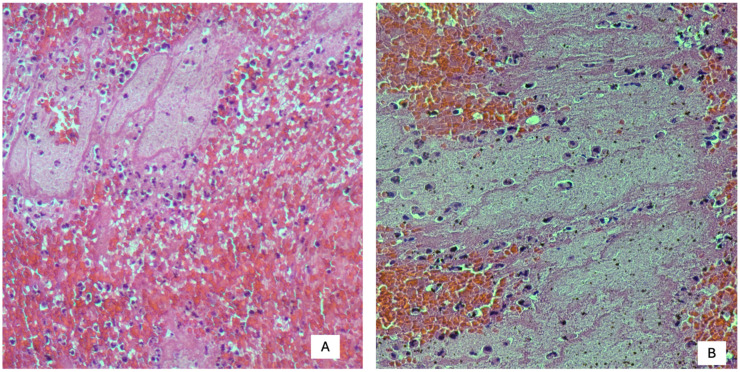
(**A**,**B**) Inflammatory infiltrate in the context of vital thrombi (20× magnification). Polymorphonuclear leukocytes are clearly identifiable within this thrombus (40× magnification).

The histological evolution of a thrombus follows a dynamic biological pathway, in which cellular elements and the extracellular matrix gradually transform over the course of days and weeks. In the initial phases, the leukocyte component is dominated by neutrophils, which represent one of the first indicators of the thrombus’ “youthfulness.” These gradually begin to decline, giving way to macrophages, which become predominant around the seventh day. During this same period, fibroblasts and fibrocytes appear, and collagen deposition begins, which are morphological signs of incipient organization (Figure 3A,B).

In the subacute phase (2–6 weeks), the repair processes become clearly evident: the macrophage infiltrate stabilizes, collagen deposition becomes widespread, and new vessels and recanalization channels begin to appear, marking the actual organizational phase of the thrombus. Beyond two months, the thrombus takes on fibrotic and cicatricial characteristics, with marked hyalinization, reduced cellularity, and a clear predominance of connective tissue, defining the late phase [18].

This pattern, inevitably modulated by interindividual variability and marked by intrathrombotic heterogeneity, has been confirmed both in thrombi removed in vivo and in pulmonary emboli. These studies have highlighted compositional gradients rich in erythrocytes/fibrin/platelets and different temporal patterns of reorganization—such as the formation of polyhedrocytes due to clot contraction—with diagnostic and forensic implications [15,33,34,35].

From a historical perspective, many authors have attempted to define chronological models for the histological dating of thrombus, based on the most frequently observed microscopic findings. The first systematic attempt dates back to Baranga [36] in 2023; a subsequent contribution was provided by Cao [37] in 2021. However, the most solid foundations for a true chronological subdivision derive from the experiments of Yuriditsky [33] in 2022. In the following years, further refinements were made by the studies of Olsen [38] and Hendley [34] in 2021. The combination of these works allowed the histological transformation of thrombus to be divided into six phases over an overall time span of approximately twelve months. Despite the descriptive accuracy of this chronological structure, authors such as Christensen [39] and Knight [40] have urged caution in applying excessively detailed schemes in forensic settings, where the need for certainty requires more robust and reproducible models. For this reason, the most widely used classification is that proposed by Fineschi et al. [14], which distinguishes three main phases considered forensically reliable.

According to this model, Phase 1 (days 1–7) is characterized by the formation of a platelet “plug” associated with fibrin deposition, with stratified growth (Zahn lines) and the absence of endothelial reaction. In this phase, the integrity of the erythrocytes is associated with early signs of leukocyte pyknosis; the von Kossa reaction may highlight calcific precipitations, while monocytes show enlarged nuclei.

Phase 2, which can last up to eight weeks, is characterized by the beginning of organization: endothelial germination and fibroblast penetration appear, siderophages are observed, the fibrin network tends to coalesce, and endothelial proliferation is observed on the surface of the thrombus.

Finally, Phase 3 includes thrombi with more than two months of evolution and shows complete hyalinization, the formation of sinuous central cavities, poor cellular representation, and recanalization through large blood-filled neovessels [14].

Despite being a fundamental tool, this classification has intrinsic limitations. The thrombotic process, in fact, does not follow a rigidly linear course but is influenced by numerous variables, such as individual comorbidities, anticoagulant therapies, a systemic inflammatory state, the thrombus localization, its size, or the involvement of venous or arterial circulation. Histological interpretation must therefore be cautious and always integrated with clinical and circumstantial data.

Subsequent studies conducted by Maffeis and Mansueto [13,18] have attempted to make the classification more objective, identifying key cell populations (macrophages, fibroblasts, fibrocytes) and correlating their presence with more clearly defined temporal intervals (Table 1).

## 4. Histochemical and Immunohistochemical Study

Over the last two decades, in addition to consolidating histomorphological criteria for assessing thrombus age, the literature has proposed several immunohistochemical panels capable of selectively “marking” specific phases of thrombus organization (neutrophils and NETs, CD163+ macrophages, matrix metalloproteinases (MMPs), inflammatory mediators such as IL-6, the urokinase-type (uPA)/tissue-type plasminogen Activator (tPA)/plasminogen activator inhibitor-1 (PAI-1) fibrinolytic pathways, cellular reprogramming signals such as autophagy and, more recently, aquaporins) in murine models and human autopsy series [15,18,19,41,42].

As previously discussed, the evolution of a venous thrombus follows a biological trajectory characterized by partially overlapping phases: neutrophil recruitment and acute cytokine response, activation of the fibrinolytic system and metalloproteinases, macrophage transition with erythrocyte removal and collagen deposition, appearance of myofibroblasts, neovascularization and recanalization, and finally fibrous maturation and resolution with pro-resolving signals. Each of these stages leaves a relatively reproducible immunohistochemical signature which, when interpreted as a panel rather than through isolated markers, allows a more robust forensic temporal inference.

Over the years, there have been numerous attempts to define the criteria useful for postmortem dating of thrombi. A contribution to immunohistochemical determination of the age of human thrombi/emboli in forensic pathology is attributable to Fineschi et al. [14], who used histochemical markers such as anti-fibrinogen, anti-platelet (CD61), anti-leukocyte (CD45), anti-neutrophil (CD15), and anti-macrophage (CD68) antibodies on thrombi collected from human cadavers. By comparing immunohistochemical findings with histological staging obtained using stains such as hematoxylin–eosin (HE), trichrome stains, and von Kossa stain, three phases of thrombus modification were identified. These phases have subsequently been refined by multiple authors, with the aim of more accurately identifying morphological, structural, and quantitative cellular changes involved in thrombus evolution [2,8,14,15,18,43,44,45,46,47,48,49,50,51,52,53].

Phase 1, comprising the first seven days after onset, is characterized by marked positivity for the platelet antigen CD61 in the absence of endothelial reaction. Leukocytes identified by CD15, CD45, and CD68 show signs of pyknosis and nuclear enlargement. Phase 2, between the second and eighth weeks, shows signs of fibrin organization detected by anti-fibrinogen antibodies, with trapping of CD15-positive neutrophils. A marked increase in CD68-positive macrophages, platelet aggregation (CD61), and scattered nuclear debris of white blood cells (CD45) is also observed. Phase 3, from the third month onwards, is characterized by compact, hyaline, fiber-rich connective tissue and a poorly represented leukocyte population (CD45 and CD68).

Starting in 2009, Nosaka et al. [43,44,45,46,47,48,49,50,51,52] initiated a line of studies aimed at developing a more coherent experimental framework in stasis-induced murine DVT models, illustrating the dynamics of cells and proteins within the thrombus and proposing qualitative and quantitative cut-offs translatable into autopsy practice. Subsequently, Furukoji et al. [41] conducted an immunohistochemical study designed to “date” aspirated thrombi in living subjects without pulmonary embolic complications. The markers used in this work allowed them to highlight fibrin aggregates, platelets (integrin α2bβ3), new vessels (α-smooth muscle actin, smooth muscle actin (SMA), and CD34), erythrocytes (glycophorin A), and macrophages (CD68, CD163, and CD206).

Using the Spearman correlation coefficient, the authors found that the number of erythrocytes (glycophorin A-positive cells) negatively correlated with time since onset, suggesting that this marker plays an important role in identifying the acute phase. Conversely, CD68- and CD163-positive macrophages showed a positive correlation with thrombus age. CD163+ macrophages, in particular, due to their hemophagocytic role and stimulation of mesenchymal stem cell growth, were highly represented in erythrocyte-rich areas and in regions containing SMA- and CD34-positive cells, thereby confirming macrophages as markers of the clot organizational phase [2].

The case report by Maffeis et al. [13], despite the limited heterogeneity of the study, clearly demonstrated the importance of comparing histological and immunohistochemical findings for accurate temporal assessment of the process. In that case, samples from the left femoral vein, lung tissue, and right ventricle were stained with hematoxylin–eosin and analyzed immunohistochemically using antibodies against CD34 (new vessels), CD68 (macrophages), and myeloperoxidase (MPO, neutrophils). The DVT sample, which was CD34-positive, showed evidence of endothelial budding; the N/M ratio derived from MPO and CD68 positivity was <1. The free surface of the thrombus and the absence of recanalization allowed the lesion to be classified within phase 2 of Fineschi’s staging [14]. Lung samples, however, despite a discrepancy between the N/M ratio of the pulmonary trunk and smaller pulmonary vessels, showed no CD34 reactivity and minimal hemorrhagic deposits on Perls staining. These findings supported a more recent onset, attributable to phase 1 according to Fineschi et al. [14].

Finally, in the study by Mansueto et al. [17], immunohistochemical analysis was applied to pulmonary emboli collected from 30 subjects, using specific markers for inflammatory infiltrates: leukocytes (CD45), T lymphocytes/monocytes (CD3), and macrophages (CD68). Further analyses were conducted by immunofluorescence using antibodies against factor VIII and fibrinogen, and Sirius Red/Fast Green histochemical staining was performed to demonstrate collagen. Immunohistochemistry did not detect lymphocytic (T-cell) infiltration within one hour. Lymphocytes were identified starting from the “recent” thrombus category (>1 h to 24 h). In recent-to-mid thrombi, an increase in inflammatory cells and initial degradation with progressive recruitment of histiocytes and fibroblasts was observed. Due to leukocyte lysis and subsequent enzyme release, which digest and destroy erythrocytes and platelets, fibrin and cellular debris (homogenization) were found to be predominant. Fibroblasts and fibrosis were located peripherally in the vascular tissue during the first hours and up to the third day, whereas they were centrally located and abundant by the fourth day. In older thrombi, proliferation of endothelial cells, increased fibrosis, and recanalization phenomena were observed.

The novelty of this study lay in the attempt to establish even tighter time ranges for determining the age of fatal PE, by using a three-point semiquantitative score for inflammatory cellular infiltration and fibrosis. Application of this score led to the development of a five-phase algorithm. The first phase (first 60 min) showed no lymphocytic (CD3) infiltration on immunohistochemistry, but intense platelet accumulation with poor association with erythrocytes on immunofluorescence. The second phase (within 24 h) showed minimal CD3 positivity within the thrombus. The third phase (24–48 h) was characterized by an increase in cellular infiltrate associated with initial cellular degradation at the thrombus periphery and recruitment of macrophages and fibroblasts. Lymphocytic infiltration and increased fibrosis defined the fourth phase (48–72 h). In the fifth and final phase, after 72 h, proliferation of endothelial cells and initial recanalization of the thrombus were observed [2].

Despite these advances, the authors noted a high risk of error in immunohistochemical investigations on cadaveric samples, partly due to the loss of antigenicity in postmortem tissues. They therefore recommended a primarily histological approach, followed by immunohistochemical techniques to increase sensitivity [2].

In light of the above, the following sections integrate the results of the studies included in this review, analyzing the cells and markers directly involved in thrombus formation and subsequent resolution in the different stages of thrombus evolution.

### 4.1. Very Early Phase (First 24–72 h)

#### 4.1.1. Neutrophils (MPO) and Macrophages

In the days and weeks following formation, thrombi may undergo propagation, embolization, dissolution, and organization with eventual recanalization. During organization and recanalization, thrombi induce inflammation and fibrosis and may either re-establish vascular flow or become incorporated into a thickened vascular wall. Several lines of evidence have shown that leukocytes, particularly neutrophils and macrophages, are intimately involved in all four processes [8].

In the first few days, the thrombus is dominated by neutrophils, whose temporal relevance is linked to their rapid increase followed by a decline that coincides with the rise in the macrophage component. According to Nosaka et al. [8], the neutrophil/macrophage (N/M) ratio in thrombi is a useful parameter for estimating thrombus age. In their study, five microscopic fields (two central and three peripheral) per section at ×1000 magnification were evaluated, showing that in the 1- and 3-day-old groups most specimens had N/M ratios > 2.0, whereas after more than 5 days no thrombus showed N/M ratios > 2.0. These findings suggest that an N/M ratio > 2.0 indicates a thrombus age of 1–3 days.

Moreover, in the 1-day-old group, four of five specimens had ratios > 5.0 (range 4.8–9.0), while in 3-day-old thrombi the N/M ratio ranged from 1.7 to 4.2. An N/M ratio markedly greater than 5.0 therefore strongly indicates an age of around 1 day. No significant differences in N/M ratios were observed between the 5–21-day groups; ratios ≤ 1.0 likely indicate an age > 5 days [8]. The N/M ratio thus represents one of the earliest indicators of thrombotic “youth” [2].

#### 4.1.2. NETs (Citrullinated Histone H3, MPO–DNA)

Platelet aggregation has traditionally been viewed as the main scaffold in thrombus development; however, accumulating evidence emphasizes the critical role of neutrophil extracellular traps in venous thrombosis, possibly providing a structural scaffold for platelet aggregation and deposition of coagulation factors. NETs are generated by activated neutrophils (recognized by MPO) and chromatin decondensation [54]. In this process, the expression of tissue factor (TF) is also induced on the surface of activated neutrophils, initiating thrombin generation and thus activating the coagulation cascade. This cascade leads to platelet activation, red blood cell aggregation, fibrin formation, and venous thrombosis. NETs thus contribute to all phases of thrombus formation, extension, and recanalization.

The NET-specific marker citrullinated histone H3 (CitH3) has significant forensic value for estimating thrombus age. By monitoring temporal changes in thrombus content and morphology, CitH3 facilitates qualitative assessment in sudden death cases attributable to DVT. According to Brill et al. [55], NETs are predominantly located in erythrocyte-rich (red) regions of thrombi and play a key role in their early formation. In the study by Pan et al. [7], five microscopic fields (two central and three peripheral) per section at ×1000 magnification were examined. Red thrombi formed within the first 3–6 h after ligation were found to play a critical role in thrombus extension. According to immunofluorescence analysis, NETs were consistently expressed after 24 h and gradually accumulated, moving from the thrombus’s center toward the vessel wall. This spatial redistribution likely reflects dynamic interactions between NETs and damaged venous endothelium, underscoring their role in thrombus stabilization and progression [55].

Temporal changes in CitH3-positive cells were then analyzed using Western blot and immunofluorescence. Thrombi collected from 3 to 6 h after ligation had too little CitH3 to be reliably detected by Western blot. CitH3 first became detectable at 24 h after ligation, increased further to peak at day 3, and then slowly decreased between days 5 and 7. By day 21, the CitH3 signal had become very faint, consistent with the resolution phase of thrombus organization. As MPO expression is a marker of neutrophil activation, performing its assessment simultaneously demonstrated that from 3 h to 1 day after ligation, MPO-positive cells increased more rapidly than CitH3-positive cells, indicating that the activation of neutrophils precedes NETosis in early thrombus formation. The shift from neutrophil activation to NET formation and stabilization was reflected in the significant drop in MPO levels by day 3, which fell below CitH3 levels. Comparative analysis of MPO-positive and CitH3-positive cells thus showed distinct temporal patterns: MPO predominated in the earliest stages, while CitH3 persisted longer [7,55].

Analysis of the CitH3/MPO ratio revealed that a ratio of approximately 1 corresponded to a thrombus formation time of around 5 days. Smaller variations in the CitH3/MPO ratio across samples were associated with shorter formation times, whereas a ratio > 2 or greater variability between samples suggested a formation time > 7 days. Overall, NETs and related biomarkers have demonstrated particular utility in assessing thrombus maturation, especially for distinguishing thrombi ≤ 72 h old from more organized lesions, thus providing a valuable tool for forensic applications. However, their persistence may be influenced by infections/sepsis and anticoagulant treatment [7].

#### 4.1.3. IL-6 and Macrophage-Derived Cytokines

Macrophages recruited during thrombus formation can produce a wide array of cytokines and growth factors, such as IL-6. In the study by Nosaka et al. [45], intrathrombotic gene expression of IL-6 increased after inferior vena cava (IVC) ligation and IL-6 exerted a negative role in thrombus resolution through the suppression of intrathrombotic proteinases. One day after IVC ligation, IL-6+ cells were present in three of five samples; thereafter, they were steadily present in all thrombi ≥ 3 days old.

The authors then analyzed intrathrombotic IL-6/macrophage (IL-6/Mϕ) ratios in five microscopic fields at ×1000 magnification, as IL-6 was mainly expressed by intrathrombotic macrophages. All samples with thrombus age ≤ 5 days had IL-6/Mϕ ratios < 0.5. In contrast, thrombi aged 7–21 days had IL-6/Mϕ ratios > 0.5. These observations imply that IL-6/Mϕ ratios > 0.5 strongly indicate a thrombus age ≥ 7 days, whereas ratios < 0.5 suggest an age ≤ 5 days [45]. Within the thrombus formation/resolution process, intrathrombotic IL-6 can therefore be considered an early phase marker, whose attenuation accompanies thrombus organization.

### 4.2. Early–Intermediate Phase (3–10 Days)

#### 4.2.1. Cellular Sources of MMP-2 and MMP-9

Collagenolysis is essential for thrombus resolution. Among the various collagenolytic enzymes, MMP-2 and MMP-9 are presumed to play a key role in collagen turnover during this process. In a study by Nosaka et al. [47], the number of MMP-9-positive cells was higher than that of MMP-2-positive cells at the analyzed time points, with a significant difference in thrombi < 7 days old.

By examining five high-power microscopic fields (×1000), the mean MMP-9/MMP-2 ratio was >2.0 (range 2.1–7.0) in all thrombi aged 1–5 days. This suggests that an MMP-9/MMP-2 ratio clearly above 2.0 strongly indicates an age ≤ 5 days. Beyond 7 days, the ratio dropped below 2.0 (0.6–1.9), implying that an MMP-9/MMP-2 ratio < 2.0 likely indicates an age > 7 days [47].

Nosaka’s work therefore shows that MMP-9 (gelatinase B) predominates in very early stages, in keeping with its action on “soft” matrix components (fibrin, gelatin), whereas MMP-2 (gelatinase A) increases with advancing organization, paralleling collagen remodeling and recanalization. A “MMP-9-high/MMP-2-low” pattern suggests an age ≤ 5–7 days, while progression toward higher MMP-2 levels supports an intermediate window [47].

#### 4.2.2. Appearance of Hemosiderin

Macrophage recruitment is essential for thrombus formation and evolution, not only because of cytokine and growth factor production but also because macrophages phagocytose red blood cells; hemoglobin is then converted to hemosiderin, which can be detected by Perls staining. Nosaka et al. [52] found that Perls-positive cells, assessed in five high-power fields (two central and three peripheral, ×1000), were inconsistently present at 3 days (1/5 samples), but consistently present at ≥5 days. This finding signals the onset of erythrocyte degradation and iron recycling. Diffused hemosiderin deposition therefore supports a temporal window of ≥5 days [15,52].

#### 4.2.3. Collagen

Increased intrathrombotic collagen content is a key event in venous thrombus organization. Myofibroblasts are the main cellular source of collagen; accordingly, collagen accumulation in thrombi closely parallels the intrathrombotic increase in myofibroblasts. Nosaka et al. [52] documented, using Masson’s trichrome staining, that collagen content increased from approximately 20% at 5 days (starting from the most peripheral thrombus regions) to >80% at 21 days, demonstrating the transition from the inflammatory phase to fibrous maturation.

#### 4.2.4. Fibrinolytic System (uPA, tPA, PAI-1)

Urokinase-type plasminogen activator (uPA) and tissue-type plasminogen activator (tPA) are trypsin-family serine proteases produced by macrophages and essential to the intrinsic coagulation system. During venous thrombus resolution, tPA is mainly involved in fibrinolysis, whereas uPA mediates cell migration and tissue remodeling. Both enzymes cleave plasminogen, a serum β-globulin deposited on fibrin filaments within the thrombus. Plasminogen activator inhibitor type 1 (PAI-1), a serine protease inhibitor, regulates uPA and tPA by inhibiting their proteolytic activity.

The time-dependent behavior of these three molecules allows us to distinguish thrombi that are still “plasmacytic” (with active uPA/tPA) from more organized phases, in which equilibrium shifts toward inhibition [48]. In a study by Nosaka et al. [48], five microscopic fields (two central and three peripheral) per section at ×1000 magnification were evaluated. The presence of uPA- and tPA-positive cells indicated thrombus ages of ≥5 and ≥7 days, respectively, while intrathrombotic PAI-1 expression indicated an age ≥ 3 days. In all thrombi aged 10–24 days, uPA/PAI-1 and tPA/PAI-1 ratios were >0.1 and >0.2, respectively, whereas in 1–7-day-old thrombi, uPA/PAI-1 ratios were <0.1 and tPA/PAI-1 < 0.2. These findings imply that uPA/PAI-1 > 0.1 and tPA/PAI-1 > 0.2 indicate an age of 10 days or more.

Furthermore, in four of five 10-day-old samples, uPA/PAI-1 ratios were <0.3, and in the remaining sample the ratio was 0.32. All thrombi aged 14–21 days had values > 0.3. These data suggest that uPA/PAI-1 ratios significantly > 0.3 strongly indicate an age > 14 days. Overall, the balance between tPA/uPA and PAI-1 influences thrombus fate: in the early-to-mid window, uPA/tPA expression increases within the thrombus, indicating active remodeling, while PAI-1 modulates the duration of the proteolytic phase.

### 4.3. Intermediate Phase (5–14 Days)

#### 4.3.1. Intrathrombotic New Vessels (CD31/PECAM-1)

Intrathrombotic recanalization via neovascularization contributes to blood flow restoration. The area occupied by intrathrombotic new vessels increases in a time-dependent manner. CD31-positive new vessels first appear at 5 days at the thrombus periphery in one of five specimens and are present in all samples after 10 days, representing a reliable histological “clock” of recanalization [52].

#### 4.3.2. Myofibroblasts (α-SMA)

In the study by Nosaka et al. [52], myofibroblasts, assessed with anti-α-SMA monoclonal antibodies, appeared at day 7 (in 3/5 samples) and increased steadily at ≥10 days, indicating the transition toward healing and thrombus contraction. When α-SMA positivity is widespread, it is possible to infer a thrombus age of ≥7–10 days.

In a subsequent study, Lee et al. [56] demonstrated that 2–4-week-old thrombi can undergo dramatic remodeling from a fibrin-dominant to a collagen-dominant microstructure, accompanied by a marked increase in stiffness. Preliminary mechanical tests showed that 1-week-old thrombi were too soft to allow reliable uniaxial measurements, whereas data at 2 and 4 weeks revealed significant changes in both composition and stiffness. Two-week thrombi consisted primarily of fibrin, with some collagen fibers near the periphery, whereas 4-week thrombi had high collagen content throughout the cross-section. Infiltration of α-SMA-positive smooth muscle cells, presumably myofibroblasts, in peripheral regions at 2 weeks and throughout the thrombus at 4 weeks strongly suggests a primary role for these cells in collagen synthesis and organization during inward radial remodeling.

#### 4.3.3. Fibrocytes (CD45+/Collagen I+)

Fibrocytes are bone marrow-derived mesenchymal progenitors that coexpress hematopoietic antigens (CD45) and fibroblast products such as type I collagen (Col I) [44]. Recruited from the bone marrow under the guidance of chemokine systems, fibrocytes constitutively produce extracellular matrix components and enzymes and can further differentiate into myofibroblasts under permissive microenvironmental conditions in vitro and in vivo. They may participate in multiple inflammatory diseases, be recruited to damaged organs and play an important role in thrombus resolution via myofibroblast transformation and neovascularization.

The time-dependent progression of myofibroblasts is very similar to that of fibrocytes, supporting the hypothesis that some myofibroblasts derive from fibrocytes [44]. In the study by Nosaka et al. [50], intrathrombotic fibrocytes were examined in murine samples by immunohistochemical detection of CD45-positive cells and type I collagen. The total number of fibrocytes was assessed in sections at ×400 magnification, and Masson’s trichrome staining was used to detect collagen accumulation. Collagen deposition was expressed as a percentage of total thrombus area.

Fibrocytes were never observed at 1, 3, or 5 days after IVC ligation. Their appearance was first detected in marginal thrombus areas from day 7, with gradual spread thereafter. In all 7-day samples, fibrocyte counts were <10; at ≥10 days after ligation, fibrocyte counts exceeded 10, peaking at 14 days and gradually declining by 21 days. Based on this qualitative and quantitative profile, fibrocytes can be considered bridging markers between inflammation and fibrosis [44].

#### 4.3.4. Endothelial Progenitor Cells (EPCs)

Endothelial progenitor cells (EPCs) contribute to neovascularization, a common process in tissue repair and tumor growth. Nosaka et al. [43] examined intrathrombotic EPC patterns by detecting CD34- and Flk-1-positive cells, defining EPCs as CD34+/Flk-1+ cells. EPCs were assessed semiquantitatively in all intrathrombotic fields at ×400 magnification.

No intrathrombotic EPCs were observed 1 and 3 days after IVC ligation [43]. EPCs appeared in all thrombi starting at 5 days, with initial detection in marginal thrombus areas. In all 5-day samples, EPC counts were <10, whereas at 7, 10 and 14 days, all samples showed EPC counts ≥ 10, with a gradual decrease by 21 days [43]. These observations indicate that EPCs accumulate in a time-dependent manner. Comparing the formation of intrathrombotic neovessels and EPC appearance, the authors found similar serial expression patterns, suggesting that EPC accumulation contributes to thrombus formation and resolution via EPC-derived neovascularization [43]. The intrathrombotic appearance of EPCs, together with the presence of CD31-positive neovessels and MMP-2 expression in thrombi beyond the “young” phase, thus accompanies recanalization and characterizes the intermediate organizational phase of thrombus evolution [43].

#### 4.3.5. Heat Shock Proteins (HSP27 and HSP70)

HSPs are a family of proteins that can be induced by various proteotoxic stresses, including high temperature, hypoxia, hyperoxia, UV exposure, nutrient deficiency, and other stimuli. Their primary role is to maintain cellular homeostasis and promote cell survival. HSP27, one of the members in the HSPB family encoded by the HSPB1 gene, is constitutively expressed in most mammalian cells and prevents aggregation of partially unfolded proteins, protects against oxidative stress by scavenging ROS, and inhibits apoptosis under stress through interference in cytochrome c release and caspase-3 activation. HSP70, a member of the HSPA family, performs chaperone functions, preventing protein aggregation and abnormal ubiquitin redistribution [50].

Nosaka et al. [50] have recently reported increased intrathrombotic expression of both HSP27 and HSP70 from days 5 to 10, suggesting that their expression was initiated by IVC ligation and the resulting blood coagulation. Intrathrombotic HSPs were mainly expressed in F4/80+ macrophages, which could promote thrombolysis and may contribute to intrathrombotic neovascularization. Although the period of their expression was rather short (5–14 days), HSP27 showed a particularly marked expression on day 10 and thus can be used as a marker for estimating thrombus age, particularly in subacute and chronic phases [50]. Thienel et al. [57] recently investigated several antithrombotic models in platelets from hibernating brown bears. Among the molecules studied, HSP47, an intracellular chaperone essential for collagen production, showed a marked reduction. Decreased or absent HSP47 led to inhibition of immune cell activation and NET formation, contributing to the prevention of blood clotting in bears, patients with spinal cord injury, and under cold conditions [57]. HSP47 has also been reported on the platelet surface, where it interacts with collagen, stabilizes platelet adhesion, and promotes thrombus formation and hemostasis [58]. In vivo, pharmacological inhibition of HSP70 in human whole blood prevented platelet aggregate formation on collagen under shear conditions. This is in agreement with the findings of Nosaka et al. [50], who demonstrated that inhibitors against HSP70 and HSP27 decrease thrombus mass in mouse models. Taken together, these data suggest that modulation of HSPs may represent a novel protective mechanism against the development of a thrombus.

### 4.4. Intermediate–Late Phase (≥10–21+ Days)

#### 4.4.1. Macrophages (CD68) and “Scavenger” Polarization (CD163)

As previously mentioned, the neutrophil/macrophage ratio (N/M) is one of the earliest indicators of thrombotic “youth” [2]. In the first days, thrombi are dominated by neutrophils, but their subsequent decline coincides with the emergence of the macrophage component that characterizes more advanced stages. In the study by Furukoji et al. [41], based on aspirated thrombi from living subjects, the number of CD163-positive cells was positively correlated with time since onset, whereas the glycophorin A-positive area was negatively correlated with time. Most macrophages expressed CD163 and were closely distributed in areas rich in erythrocyte-positive cells, CD34, and SMA.

CD163 is a scavenger receptor for the hemoglobin–haptoglobin (HbHp) complex in macrophages. Its expression is influenced by several microenvironmental factors: IL-6, IL-10, and glucocorticoids increase CD163 expression in monocytes/macrophages, whereas tumor necrosis factor-α, IL-1β, interferon-γ, lipopolysaccharide, IL-4, IL-13, oxidative stress, and hypoxia decrease it [41]. High concentrations of the HbHp complex also induce CD163 expression with increased secretion of IL-6 and IL-10 [41]. Hemoglobin released from lysed erythrocytes in venous thrombi likely increases HbHp complex formation and upregulates CD163 on thrombus-associated macrophages. Previous studies have suggested that HbHp–CD163 binding supports anti-inflammatory macrophage responses [41].

Macrophages participate in the resolution of inflammation and the mediation of tissue repair and remodeling through the production of anti-inflammatory cytokines and angiogenic factors, matrix digestion, and deposition. Thus, interactions between CD163+ macrophages, endothelial cells, and myofibroblasts/smooth muscle cells may provide evidence for the role of CD163+ macrophages in venous thrombus resolution and organization [41]. CD163 expression, together with the decrease in the erythrocyte (e.g., glycophorin A-positive) component, demonstrates the transition from “red” to “white/fibrous” thrombus and may indicate a later organizational phase [41].

#### 4.4.2. Aquaporins (AQP-1, AQP-3)

Aquaporins (AQPs) are membrane channel proteins involved in water transport, widely distributed in most tissues. Thirteen AQPs have been identified in mammals. They participate in multiple physiological processes (urine concentration, body fluid homeostasis, brain function, glandular secretion) and pathological mechanisms (cancer progression, angiogenesis, etc.) [49]. AQP-1 is widely expressed in various organs, tissues, and cells, including red blood cells, proximal tubular cells, lungs, secretory glands, skeletal muscle, and peritoneum, but shows limited distribution in the central nervous system [49]. AQP-1 also contributes to platelet coagulation function [59].

AQP-1 positivity has also been widely observed in erythrocytes and dermal capillaries, supporting the role of AQP-1 in the process of angiogenesis, as suggested in the study by Nosaka et al. [49]. AQP-3 expression was documented in intrathrombotic macrophages, suggesting a possible role for AQP-3 in facilitating macrophage migration. Both AQP-1 and AQP-3 are reportedly involved in both thrombus formation and resolution and may serve as useful markers for estimating the age of a thrombus [42,49].

Temporal changes in intrathrombotic AQP-1 expression were inversely proportional to intrathrombotic collagen accumulation. Collagen deposition, visualized as blue areas in Masson’s trichrome-stained sections, was quantified relative to total thrombus area, while brown AQP-1–positive areas were expressed as a percentage of thrombus area. A larger AQP-1+ area corresponded to a smaller collagen area and was associated with thrombus ages ≥ 10 days [49,60]. AQP-1-positive areas initially exceeded 70%, decreasing to <50% seven days after vessel ligation and to 11% at 21 days [49,60].

A count of >30 AQP-3+ cells indicates a thrombus age of 10–14 days. AQP-3 positivity first appeared in peripheral thrombus regions 3 days after ligation, then progressively increased, peaking at day 10 [49,60]. Given that AQP-3 is expressed by a subset of intrathrombotic macrophages, the ratio between AQP-3+ macrophages and total macrophages may be useful in age estimation. AQP-3+ cells and F4/80+ macrophages were semiquantitatively assessed in five high-power fields (×1000). A ratio < 50% indicated a thrombus age ≤ 7 days.

Overall, AQP-1/AQP-3 expression evolves in parallel with thrombus organization and recanalization, positioning these aquaporins as markers of intermediate–late phases. Dedicated reviews highlight their promising applicability when used in combination with “classic” markers [42,49,60].

#### 4.4.3. Autophagy (LC3, p62)

One important biological mechanism that breaks down damaged cellular components is autophagy. Autophagy can be evaluated using biochemical and immunochemical markers such as p62 and microtubule-associated protein 1a/1b-light chain 3 (LC3). Intrathrombotic CD68+ macrophages in several human thrombus samples from forensic autopsies displayed increased LC3 and p62 levels in a study by Nosaka et al. [51], which is consistent with results in murine thrombi. These results suggest that macrophage autophagy is suppressed during thrombus formation. Because autophagy suppression can result in abnormal p62 accumulation, which can activate NF-κB and exacerbate inflammatory responses, it may be connected to macrophage activation, an essential step in thrombus formation and resolution. After IVC ligation, LC3+ cells—which are necessary for the formation of autophagosomes—were found in all thrombi one day or more later. they declined after three days and subsequently increased again for ten days. On the other hand, p62+ cells were barely noticeable one day but were present three days later [51]. The relationship between thrombus age and the colocalization of LC3 and p62 in intrathrombotic F4/80+ macrophages was examined using dual-color immunofluorescence. A thrombus age of 5–10 days was associated with the presence of ≥10 cells that were positive for both LC3 and p62 in five high-power fields (×1000) [51].

Comparing LC3- and p62-positive cells revealed that all 1-day-old thrombi had p62/LC3 ratios < 0.5, while 29 out of 30 thrombi aged 3–21 days had a p62/LC3 ratio > 1.0. Thus, thrombus ages of ≥3 days and approximately 1 day are indicated by ratios > 1.0 and < 0.5, respectively. A thrombus age of roughly 14 days is indicated by a ratio > 2.0 [51]. Therefore, the LC3/p62 panel adds robustness, especially when evaluating the intermediate–late stage of thrombus dating.

#### 4.4.4. FPR2/ANXA1 (Pro-Resolving Signals)

Formyl peptide receptor 2 (FPR2), a member of the formyl peptide receptor (FPR) family of chemoattractant receptors, is a multi-ligand receptor long implicated in inflammation regulation. Endogenous FPR2 agonists include annexin A1 (ANXA1) and its active N-terminal fragment, serum amyloid A (SAA), lipoxin A4 (LXA4), resolvin D1 (RvD1), and other peptides/proteins and lipids [61]. The N-terminal peptide Ac2–26 of ANXA1 has been shown to regulate neutrophil accumulation, macrophage polarization, and angiogenesis [62]. Since targeting the AnxA1/FPR2 pathway can inhibit thromboinflammation and cerebral thrombosis, it is plausible that FPR2 and ANXA1 participate in thrombus evolution [63].

In the study by Huang et al. [62], FPR2 was predominantly expressed by intrathrombotic neutrophils (polymorphonuclear cells, PMNs) and macrophages (mononuclear cells, MNCs), whereas ANXA1 was mainly distributed in neutrophils, endothelial cells of neovessels, and fibroblasts. Given this localization, ANXA1 can facilitate angiogenesis and fibroblast migration and suppress neutrophil accumulation, promoting neutrophil apoptosis via FPR2 activation. Apoptotic neutrophils then release ANXA1-derived peptides that induce macrophage recruitment [62].

Huang et al. [62] simultaneously evaluated FPR2 and ANXA1 expression using immunohistochemistry, Western blot, and RT-qPCR to provide more detailed and objective data for thrombus age estimation. Double immunofluorescence combining anti-FPR2 with anti-myeloperoxidase (anti-MPO, neutrophil marker) or anti-CD68 (macrophage marker) revealed that neutrophils and macrophages are the main FPR2-expressing cells during thrombogenesis. One to seven days after IVC ligation, many PMNs showed FPR2 immunoreactivity, and from day 3 onwards FPR2-positive staining was also observed in intrathrombotic MNCs, which tended to accumulate in marginal thrombus zones by day 7. As thrombus age increased, FPR2-positive cells became less detectable [62].

Morphometric evaluation of five high-power microscopic fields (×1000) showed that the percentage of FPR2-positive cells was >75% (76.89–94.61%) from 1 to 7 days after ligation; at 10 days, values ranged from 51.62% to 72.27%; at 14 and 21 days, values ranged from 19.82% to 52.98% [62]. The FPR2 protein/GAPDH protein ratio was also analyzed: values > 0.9 were associated with thrombus ages of 1–7 days. FPR2 mRNA levels appeared even more sensitive for age determination: mRNA RQ values >5.6 and >8.4 correlated with ages of 1–7 days and 5 days, respectively [62].

ANXA1-positive cells were detected throughout thrombus development. In 1–7-day-old thrombi, recruited PMNs showed ANXA1 immunoreactivity. ANXA1 staining was also observed in intrathrombotic neovessel endothelial cells from day 5 and in fibroblasts between days 10 and 21. PMNs expressing ANXA1, identified by combined anti-ANXA1 and anti-MPO immunofluorescence and Western blotting, showed significantly increased ANXA1 protein levels at 5, 7, 10, 14, and 21 days after ligation, with peak ANXA1/GAPDH protein ratios at 14 days. ANXA1 protein and mRNA expression levels were higher at 10–14 days than at other time points.

Combining these data, the authors reported that an ANXA1/GAPDH protein ratio > 1.1 together with an ANXA1 mRNA RQ value > 2.9 could identify thrombi aged 10–14 days. The FPR2/ANXA1 axis therefore increases with advancing thrombus age and correlates with organizational maturation, making it a candidate indicator of resolution [62].

At the end of this discussion, some clarifications are necessary. Most data derive from animal models, and translation to humans must be approached with caution. Furthermore, intrathrombotic heterogeneity requires extensive sampling and combined interpretation of multiple markers rather than reliance on a single parameter. Indeed, integrated data on neutrophil extracellular traps (NETs), CD163+ macrophages, MMPs, fibrinolytic pathways, IL-6, aquaporins, autophagy, and HSPs provide an increasingly comprehensive and reliable picture of thrombus evolution (Table 2).

### 4.5. Tissue Factor and Coagulation Activation

Tissue factor is a key player in the pathophysiology of venous thromboembolism and is the primary initiator of the extrinsic coagulation pathway [64,65,66,67,68]. In pathological conditions, TF can be induced in leukocytes and, to a lesser extent, endothelial cells and released into the bloodstream on procoagulant microparticles. In physiological conditions, TF is primarily expressed by perivascular cells and is separated from circulating blood [68]. Activation of endothelium and leukocytes stimulates TF expression and the accumulation of TF-positive microparticles in the context of venous stasis and hypoxia, such as in deep vein valve pockets. This can initiate and spread thrombus formation on an otherwise mostly intact venous wall [68].

A crucial role for TF in the development and expansion of venous clots is further supported by experimental models of deep vein thrombosis. Thrombi rich in fibrin and blood cells form quickly in a rat model of inferior vena cava ligation. Immunohistochemistry reveals TF-expressing leukocytes within the thrombus and TF staining on the venous endothelium, frequently co-localizing with protein disulfide isomerase [68].

Functional TF can be found within the thrombus in rabbit models of venous thrombosis, and antibody-mediated TF inhibition restricts thrombus propagation to a degree comparable to direct thrombin inhibition, suggesting that continuous TF activity is essential for clot growth [66].

Moreover, in cancer-associated thrombosis, tumor-derived microparticles bearing high levels of active TF markedly increase the incidence of deep vein thrombosis in mice and are recruited to the thrombus, in part through interactions with neutrophil extracellular traps, providing a mechanistic link between malignancy, NET formation and venous thrombosis. From a forensic perspective, these data underline the importance of TF-dependent systemic procoagulant states, particularly in immobilized or oncological patients, when reconstructing the sequence of events leading to fatal pulmonary embolism [65,67].

However, TF expression in the vein wall or within thrombi has not yet been systematically correlated with precise temporal windows of thrombus organization, so its current relevance is predominantly mechanistic rather than as a stand-alone marker for thrombus dating.

### 4.6. Platelets and Venous Thromboembolism

For a long time, platelets were thought to be important in thrombosis, but the coagulation cascade was thought to be more important in VTE [69,70,71,72,73]. It is now evident from experimental and clinical data that this perspective is overly simplistic. Under conditions of decreased flow, platelets are quickly drawn to the venous wall in animal models of deep vein thrombosis, where they actively participate in the formation and spread of the thrombus alongside neutrophils and monocytes. Specifically, platelets interact with leukocytes via receptors like GPIbα and help form NETs, which reinforce coagulation and inflammation in the developing venous clot [70].

Under conditions of decreased flow, platelets are quickly drawn to the venous wall in animal models of deep vein thrombosis, where they actively participate in the formation and spread of the thrombus alongside neutrophils and monocytes.

In particular, platelets interact with leukocytes through receptors such as GPIbα and contribute to the formation of NETs, which strengthen inflammation and coagulation in the developing venous clot [70].

In addition to being present in the thrombus, platelets tightly integrate with the coagulation system and offer a highly procoagulant surface [72]. Phosphatidylserine, which aids in the formation of coagulation complexes and the production of thrombin and fibrin, is exposed when they are activated by a number of receptors, including the collagen and fibrin receptor GPVI. Recent research has demonstrated that patients with DVT or pulmonary embolism exhibit elevated levels of circulating procoagulant platelets and platelet activation markers, and that genetic or pharmacological modification of platelet signaling pathways, particularly of procoagulant platelet subsets, can significantly reduce venous thrombus formation in experimental models [72].

From a forensic perspective, these findings underline that venous thrombi are not merely fibrin red cell clots, but contain a dynamic platelet component, especially in the early and organizing phases. Conventional histology allows recognition of platelet-rich areas, and immunohistochemistry for platelet markers can document their distribution within the clot. However, the temporal behavior of platelet markers has not yet been systematically correlated with precise time intervals of thrombus age, so their value as isolated chronological indicators appears limited. At present, platelet-related findings are best interpreted in combination with leukocyte/NET markers, macrophage infiltration, and matrix remodeling features, as part of an integrated, multilevel approach to thrombus dating.

### 4.7. Limitations

The present review is subject to some limitations. First, most experimental data on thrombus aging and biomarker dynamics derive from murine models. Although these models provide a precisely controlled temporal framework, species-related differences in vascular biology, coagulation, and inflammatory responses may limit the direct translation of these findings to humans. As a consequence, the proposed staging schemes and immunohistochemical markers should be applied while taking into account this aspect and should therefore be considered as provisional until they are accurately validated and well-characterized in humans. Secondly, the available human data are highly heterogeneous in terms of clinical context, sampling protocols, and histological/immunohistochemical techniques, which further complicates the definition of reliable time intervals. These limitations underline the need for prospective and multicenter human studies when trying to confirm and refine the temporal patterns described in the current literature.

## 5. Conclusions

In forensic pathology, dating thrombi is still a challenging task that necessitates combining the clinical and circumstantial context with macroscopic, histological, and immunohistochemical findings. A multilevel approach is required because no single marker can accurately determine the age of a thrombus. This involves a thorough autopsy with systematic sampling of pertinent vascular districts, followed by routine histology with multiple samples from various areas of the same thrombus. By differentiating between early neutrophil/NET-rich phases, intermediate stages dominated by macrophages and early organization, and late phases marked by revascularization and collagen-rich remodeling, immunohistochemistry can further clarify this interpretation. Rather than being absolute, these estimates should always be viewed as probabilistic. The validation of combined immunohistochemical panels in extensive, well-characterized human autopsy series should be the main focus of future research in order to create a standardized approach, which is required as no single marker can accurately determine the age of a thrombus. With this aim, systematic sampling of relevant vascular districts, followed by multiple samples from different parts of the same thrombus for routine histology should be performed. These efforts could help improve diagnostic precision and create more powerful tools for the forensic reconstruction of thromboembolic events.

## Figures and Tables

**Table 1:** Histologic timeline on hematoxylin and eosin (H&E): key morphologic features for age. ECM: extracellular matrix; RBC: Red blood cells.

Phase	Time Window	Key Histological Features	Practical Notes
Early thrombus	0–3 days	RBC-rich clot; prominent fibrin; platelets; neutrophils at margin; absent or minimal fibroblasts	Best window for identifying a fresh thrombus; influenced by PMI and inflammation
Intermediate thrombus	3–10 days	Macrophage infiltration; early fibrosis; granulation-like tissue; beginning of ECM remodeling	Overlap with early/late phases; interpret with other markers
Late organizing thrombus	10–21 days	Collagen deposition; fibroblast proliferation; partial recanalization; siderophages	Indicator of non-recent thrombosis; variable among vessels
Chronic/Recanalized thrombus	>21 days	Mature fibrosis; well-formed channels; neovascularization; thick collagen bundles	Distinctive of chronicity; cannot distinguish months vs. years

**Table 2:** Immunohistochemical and molecular markers for thrombus dating, grouped by temporal window and biological meaning, with corresponding strengths and limitations. VEGF: vascular endothelial growth factor.

Marker/Pathway	Diagnostic Timeframe	Biological Meaning	Model	Strengths	Limitations
MPO/CitH3 (NETs)	0–72 h	Neutrophil activation and NET formation	Mouse models; limited human data	High specificity for early thrombosis	Increase in sepsis/COVID; postmortem degradation
CD42b, vWF	≤3–5 days	Platelet activation, early thrombus consolidation	Human and animal	Good for fresh thrombi	Timing overlap with other phases
MMP-9/MMP-2 ratio	3–10 days	ECM remodeling and leukocyte infiltration	Mouse; limited human data	Good temporal discrimination	Affected by inflammation, PM interval
uPA/tPA/PAI-1	3–7 days peak	Fibrinolytic pathway activation/exhaustion	Human aspirated thrombi; animal	Correlates with evolution	Affected by therapy and systemic disease
Aquaporins (AQP1/3)	7–14 days	Cell migration and remodeling	Animal predominant	Marker of organization	Limited human validation
CD68/CD163 macrophages	>7 days	Macrophage polarization during organization	Human and animal	Robust and reproducible	Non-specific; increase in chronic inflammation
α-SMA, Collagen I/III	>10–14 days	Smooth muscle ingrowth; fibrosis	Human/animal	Strong indicator of organization	Poor specificity for precise dating
LC3/p62	10–21 days	Autophagy pathway activation	Mostly animal	Promising sequential marker	Insufficient human validation
ANXA1/FPR2	>10 days	Resolution of inflammation, reparative signaling	Animal; rare human	Biologically plausible	Experimental
Recanalization markers (CD34+, VEGF)	>21 days	Neoangiogenesis, late organization	Human autopsy	Strong marker of chronicity	No precise cut-offs

## Data Availability

No new data were created or analyzed in this study. Data sharing is not applicable to this article.
